# The effect of ice-binding proteins on the cryopreservation of *Caenorhabditis elegans*

**DOI:** 10.17912/micropub.biology.000734

**Published:** 2023-04-07

**Authors:** Masahiro Kuramochi, Tatsuya Arai, Kazuhiro Mio, Sakae Tsuda, Yuji C Sasaki

**Affiliations:** 1 Graduate School of Science and Engineering, Ibaraki University, Hitachi, 316-8511, Japan; 2 Graduate School of Frontier Sciences, The University of Tokyo, Kashiwa, 277-8561, Japan; 3 AIST-UTokyo Advanced Operando-Measurement Technology Open Innovation Laboratory (OPERANDO-OIL), National Institute of Advanced Industrial Science and Technology (AIST), Kashiwa, 277-8565, Japan

## Abstract

Ice-binding proteins (IBPs) are capable of binding ice crystals and inhibiting their growth. IBPs have also been reported to stabilize cell membranes under non-freezing conditions. The effects of IBPs help to reduce cold- and freezing-induced damage to cells and tissues in cryopreservation. Here, we examined whether certain IBPs, namely, fish NfeIBP6 and NfeIBP8 and fungal AnpIBP1a N55D (AnpIBP), improve the recovery rate of the nematode *Caenorhabditis elegans* after a deep cryopreservation at −80°C. The expression of fungus-derived AnpIBP in *C. elegans* significantly improved its recovery rate. This result provides useful information to establish a cryopreservation technique for long-term storage using IBP molecules.

**Figure 1. Recovery rates of control animals and IBP-expressing worms:**
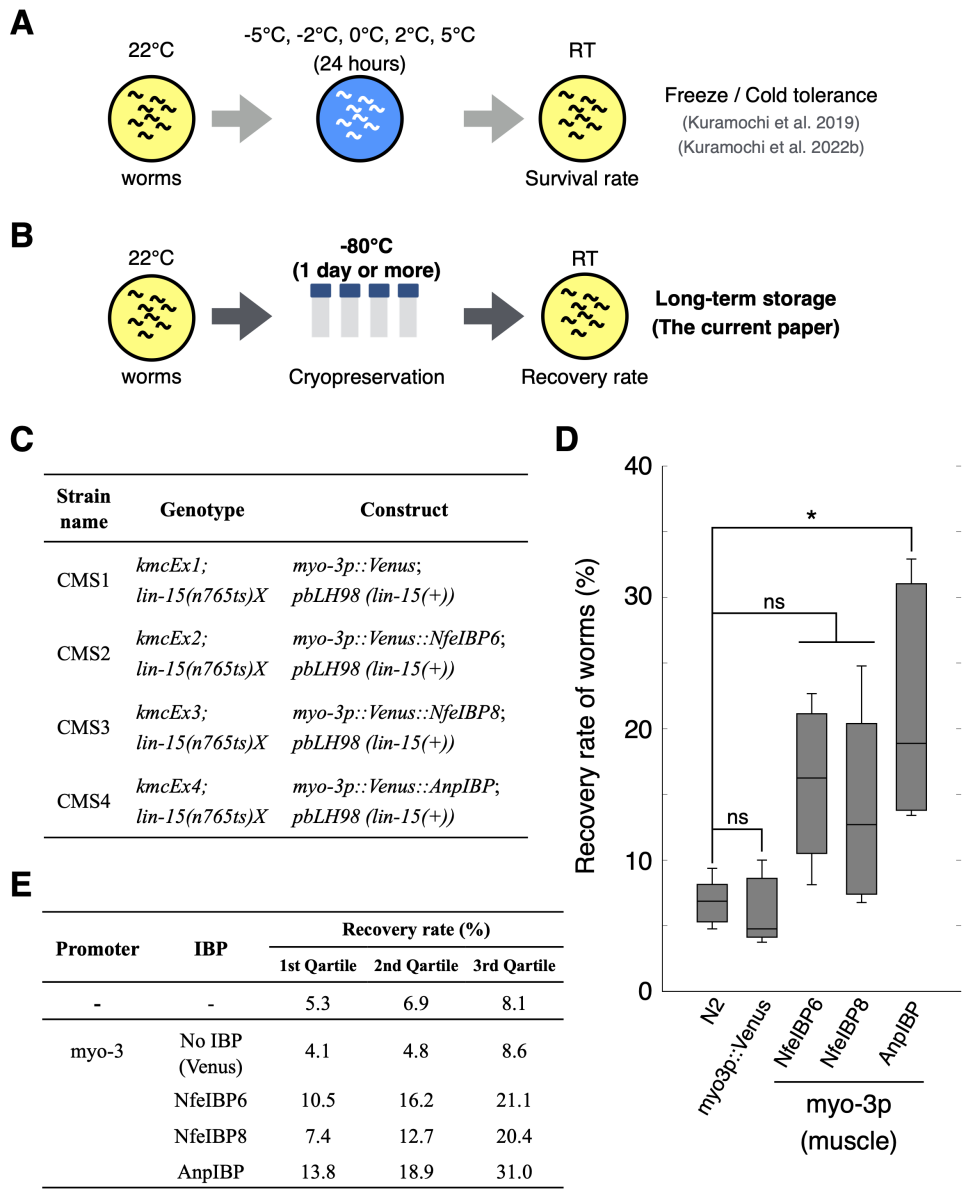
(A) Experimental procedures to observe the freeze and cold tolerance in living *C. elegans* (Kuramochi et al. 2019; Kuramochi et al. 2022b). (B) Experimental procedure to observe the recovery rate of *C. elegans* after cryopreservation. (C) Strain list. (D) Recovery rate of control animals and each IBP-expressing worm after cryopreservation at −80°C. In each assay, n > 100 (group = 5). Boxes show the first, second and third quartiles. The Wilcoxon rank sum test with Bonferroni correction was performed. *p < 0.05. (E) Detailed information on the recovery rate in (D).

## Description

Ice-binding proteins (IBPs) inhibit the growth of ice crystals by binding to them (Harding et al. 1999; Graether et al. 2000) . Ice growth inhibition is closely related to the freezing point depression activity of an IBP which is generally evaluated as thermal hysteresis (TH) value, a difference between the melting and freezing points of that solution (Knight et al. 1984; Fletcher et al. 1986) . Recently, we reported that IBPs improve the survival rate of *C. elegans* during exposure to −5 °C (freezing) ( Fig. 1A ) (Kuramochi et al. 2019) . IBP exhibiting high TH activity dramatically improved the survival rate after the freezing. Ice crystals were probably inhibited by IBPs. Our data obtained using the diffracted X-ray blinking technique suggested that IBP binds to ice crystals in the body of living *C. elegans* (Kuramochi et al. 2022a). Ice growth inhibition reduces the damage to tissues and cells of living *C. elegans* under freezing conditions. In addition, we found that IBPs also improve the survival rate after +2 °C and +4 °C hypothermic preservations (Kuramochi et al. 2022b). IBPs stabilize cell-membrane lipids at such temperatures (Rubinsky et al. 1991; Tomczak et al. 2002) . IBPs have the potential to protect tissues and cells from cold- and freezing-induced damage. We speculated that these effects are probably effective to improve the recovery of *C. elegans* after a deep cryopreservation at −80°C.

Here, we examined whether certain IBPs, namely, fish NfeIBP6 and NfeIBP8 and fungal AnpIBP1a N55D (AnpIBP), improve the recovery rate of *C. elegans* after cryopreservation at −80°C (Figs. 1B and 1C). NfeIBP6 and NfeIBP8 were isolated from *Zoarces elongatus Kner* (Notched-fin eelpout). AnpIBP was recently isolated from *Antarctomyces psychrotrophicus* . The TH activities of NfeIBP6, NfeIBP8 and AnpIBP are approximately 0 °C (0.4 mM), 0.5 °C (0.4 mM), and 0.7 °C (0.3 mM), respectively. Our previous paper showed that the survival rates of transgenic worms expressing IBPs in neurons or intestinal cells during exposure to freezing were lower than those of worms expressing IBPs in body wall muscles (Kuramochi et al. 2019) . Transgenic worms expressing IBPs in body wall muscles are therefore suitable for use to observe the recovery rates after cryopreservation. After the cryopreservation, the recovery rates of the wild-type animals and transgenic worms expressing the fluorescent protein named *Venus* in body wall muscles were 6.9% and 4.8%, respectively. In contrast, the survival rates of the worms expressing NfeIBP6, NfeIBP8, or AnpIBP in body wall muscles were 16.2%, 12.7%, and 18.9%, respectively (Figs. 1D and 1E). The recovery rate of transgenic worm expressing AnpIBP in body wall muscles was significantly higher than that of wild-type animals. IBPs presumably protected the cells from cold- and freezing-induced damage. NfeIBP6, NfeIBP8, and AnpIBP differ in TH activity but did not appear to show significant differences in the recovery rates of IBP-expressing worms. Overall, not only the inhibition of ice crystal growth but also cell membrane protection are assumed to be the key factors to improve the viability of *C. elegans* in the −80 °C-cryopreservation.

In this study, we found that IBPs expressed in living worms improve the recovery rate after cryopreservation. The mechanism of reducing cold- and freezing-induced damage to cells and tissues in cryopreservation might be related not only to the inhibition of ice crystal growth but also to cellular protection. Further investigation is needed to understand the mechanism by which IBPs achieve cryopreservation.

## Methods

Strains and constructs generated previously (Kuramochi et al., 2019) were used in this study. We used the *myo-3* promoter region for the specific expression of IBPs in body wall muscles. The region containing the promoter was amplified by PCR and inserted at the *XbaI* and *ApaI* sites of the plasmid pPD95.79 (a kind gift from Andrew Fire). To generate the transgenic animals, the plasmid DNAs were injected into the *lin-15 (n765ts)* mutant at a concentration of 30−50 ng/µL with a pbLH98 *lin-15* (+) injection marker using a standard microinjection method (Mello et al. 1991) . Worms were cultivated at 22 °C on nematode growth medium (NGM) plates with sufficient food ( *Escherichia coli* OP50). Well-fed worms were used for cryopreservation. We used 3-5 medium NGM plates with large numbers of fresh L1-L4 and adult animals. The NGM plates were washed two times with 1 mL of M9 buffer. The M9 suspension with worms was collected in conical tubes. This was performed on 3-5 plates per sample. The M9 suspension was distributed 1 mL to five cryovials, and then was mixed with 1 mL of freezing solution (Brenner 1974; Stiernagle 2006) . The cryovials were transferred to a −80 °C deep freezer. In this cryopreservation procedure, we did not use a Styrofoam box. A Styrofoam box causes slow ice crystal formation and prevents cellular damage because it slows the rate of freezing. To investigate the effect of IBP molecules on ice control, we performed cryopreservation under conditions that eliminated the slow freezing effect of the Styrofoam box. Worms were stored for 1 day or more at −80 °C. For recovery, tubes were thawed at room temperature (RT) until all ice had turned to liquid. Then, the contents of the tube were poured into a fresh NGM plate. After 1 hour at RT, the numbers of dead and living animals in the plate were counted based on worm movement. Recovery rates were obtained from five trials. Recovery rates in wild-type and IBP worms did not satisfy a Gaussian distribution based on the Shapiro-Wilk test. A non-parametric Wilcoxon rank sum test with Bonferroni correction was performed to evaluate the recovery rate using R version 3.6.1.

## Reagents

N2, and MT1642 *lin-15 (n765ts)* . Strains were provided by the CGC, which is funded by the NIH Office of Research Infrastructure Programs (P40 OD010440).
